# A repository based on a dynamically extensible data model supporting multidisciplinary research in neuroscience

**DOI:** 10.1186/1472-6947-12-115

**Published:** 2012-10-08

**Authors:** Luca Corradi, Ivan Porro, Andrea Schenone, Parastoo Momeni, Raffaele Ferrari, Flavio Nobili, Michela Ferrara, Gabriele Arnulfo, Marco M Fato

**Affiliations:** 1 University of Genoa, Dept. of Computer Science, Bioengineering, Robotics and Systems Engineering, Genoa, Italy; 2 Nextage srl, Genoa, Italy; 3 Texas Tech University, Lubbock, TX, USA; 4 Clinical Neurophysiology, Dept. Of Neuroscience, Ophthalmology and Genetics, University Hospital S. Martino, Genoa, Italy

**Keywords:** Neuroscience, Data models, Multidisciplinary studies

## Abstract

**Background:**

Robust, extensible and distributed databases integrating clinical, imaging and molecular data represent a substantial challenge for modern neuroscience. It is even more difficult to provide extensible software environments able to effectively target the rapidly changing data requirements and structures of research experiments. There is an increasing request from the neuroscience community for software tools addressing technical challenges about: (i) supporting researchers in the medical field to carry out data analysis using integrated bioinformatics services and tools; (ii) handling multimodal/multiscale data and metadata, enabling the injection of several different data types according to structured schemas; (iii) providing high extensibility, in order to address different requirements deriving from a large variety of applications simply through a user runtime configuration.

**Methods:**

A dynamically extensible data structure supporting collaborative multidisciplinary research projects in neuroscience has been defined and implemented. We have considered extensibility issues from two different points of view. First, the improvement of data flexibility has been taken into account. This has been done through the development of a methodology for the dynamic creation and use of data types and related metadata, based on the definition of “meta” data model. This way, users are not constrainted to a set of predefined data and the model can be easily extensible and applicable to different contexts. Second, users have been enabled to easily customize and extend the experimental procedures in order to track each step of acquisition or analysis. This has been achieved through a process-event data structure, a multipurpose taxonomic schema composed by two generic main objects: events and processes. Then, a repository has been built based on such data model and structure, and deployed on distributed resources thanks to a Grid-based approach. Finally, data integration aspects have been addressed by providing the repository application with an efficient dynamic interface designed to enable the user to both easily query the data depending on defined datatypes and view all the data of every patient in an integrated and simple way.

**Results:**

The results of our work have been twofold. First, a dynamically extensible data model has been implemented and tested based on a “meta” data-model enabling users to define their own data types independently from the application context. This data model has allowed users to dynamically include additional data types without the need of rebuilding the underlying database. Then a complex process-event data structure has been built, based on this data model, describing patient-centered diagnostic processes and merging information from data and metadata. Second, a repository implementing such a data structure has been deployed on a distributed Data Grid in order to provide scalability both in terms of data input and data storage and to exploit distributed data and computational approaches in order to share resources more efficiently. Moreover, data managing has been made possible through a friendly web interface. The driving principle of not being forced to preconfigured data types has been satisfied. It is up to users to dynamically configure the data model for the given experiment or data acquisition program, thus making it potentially suitable for customized applications.

**Conclusions:**

Based on such repository, data managing has been made possible through a friendly web interface. The driving principle of not being forced to preconfigured data types has been satisfied. It is up to users to dynamically configure the data model for the given experiment or data acquisition program, thus making it potentially suitable for customized applications.

## Background

Neuroscience is the study of all aspects concerning the anatomy and function of nervous system, both in health and in disease. Neuroscientists may use different data, ranging from clinical and neuropsychological evaluations and neuroimages to molecular genetics exams and blood biomarkers, to better understand (i) the development and function of nervous system in normal conditions and (ii) the dynamics of degeneration, such as in case of neurodegenerative disorders.

Combining clinical, imaging and molecular data in robust, extensible and distributed databases represents a fundamental need for modern neuroscience. Large datasets, including several data types coming from different sources and described by the associated metadata through standard formats, need to be collected, stored, monitored and analyzed. As a consequence, effective hardware and software environments must be provided to support data integration and analysis that ought to establish correlations among the totality of collected data.

According to the International Neuroinformatics Coordination Facility (INCF), neuroinformatics is the research field that encompasses the organization of neuroscience data and the application in neuroscience of computational models and analytical tools [1]. In this regard, our approach has been intended to provide a flexible and extensible data model and its implementation in a software environment has been aimed at supporting clinicians and researchers in managing multidisciplinary neuroinformatics projects.

### The data challenge

Integrating several disciplines and dealing with different information and data represent a big challenge in terms of neuroscience data management. In fact, data may range from simple textual descriptions to time series (Electroencefalography (EEG)), anatomical data (Computer Tomography (CT), Magnetic Resonance Imaging (MRI)) and functional data (Functional MRI (fMRI), Positron Emission Tomography (PET)/ Single Photon Emission Computer Tomography (SPECT)). Data size and type can vary from few Kilobytes of a textual annotation on a patient, or a psychological questionnaire, to the hundreds of Megabytes of modern MRI/CT study or all day long multichannel EEG recordings [2].

Molecular genetics and the investigation of the whole genome by means of techniques such as the whole genome sequencing, whole exome sequencing or genome wide association studies may generate an overwhelming amount of digitized data [3] that need to be managed through ever developing bioinformatics tools, at the price of more and more data, metadata, and storage space to handle. As a response to these challenges, software environments and tools must be extensible in data modalities as well as scalable in storage and computational resources.

### The information challenge

Data management in neuroinformatics needs to provide not only adequate infrastructures to store, search and retrieve large data sets, but also tools and standards for defining their structure, exploring their content and understanding their meaning. In other words, scientists need to focus on aspects such as accessibility, integration and annotation of data. Providing extensible software environments able to effectively target the rapidly changing data requirements and structures of research experiments is even more difficult. In these cases, software environments should be flexible enough to allow the dynamical definition of new types of data and metadata, to support different associations between data and metadata, to dinamically modify data models and structures, to redefine the search paths and to add distributed sources of data.

### State of the art

Several data models have been defined to describe structured data for storage in data management systems for biomedical applications. The main aim of such data models has been to provide the definition and format of data for supporting the development of suitable information systems.

XCEDE (XML-based Clinical and Experimental Data Exchange) [4] is a widespread data model providing an extensive metadata hierarchy for describing and documenting research and clinical studies. The subdivision of experimental data at various granularity levels is granted by a taxonomy of hierarchical data types. At each hierarchical level, elements contain level-specific information, whose schema may be used to store metadata specific for the experiment or related to the data modality. The linking rules between levels are flexible enough to allow the omission of other levels if the user finds them unnecessary. Due to its powerful general schema and to the inherent flexibility of its approach, the XCEDE model, describing a complete metadata hierarchy for clinical experiments, has been taken as a starting point for building the structure of our data model. Some elements at the base of the XCEDE model (projects, subjects, visits, studies, episodes and acquisitions) have been extracted and further developed in our work together with some data and metadata hierarchies used by the BIRN (Biomedical Informatics Research Network) initiative [5,6].

Also the Functional Genomics Experiment (FuGE) object model is aimed at providing a framework for the development of standards in life science. As such, FuGE provides a solid foundation for other technology-specific, life-science standards and data formats and is currently being used to develop formats for microarrays, proteomics, metabolomics and various other technologies [7]. As we had to manage also data in the field of genomics, references to FuGE have been considered during our work. From another point of view, ontologies try to define, through a common vocabulary, a structured representation of knowledge that can be used by either humans or automated software agents on a particular domain [8]. Within this work, ontologies are used to support users in defining the names of attributes of data types. This is very helpful to build data types with standard annotations. Two ontologies belonging to the Open Biomedical Ontologies (OBO) [9,10], providing a list of other useful ontologies, have been taken into account. They are: 

∙ The Ontology for Biomedical Investigation (OBI, formerly called Fugo (Functional Genomics Ontology) [11,12]

∙ The Sequence Ontology (SO) [13,14]

Several software platforms have been proposed in the last years to manage multimodality neuroscience and neuroimaging data, but just a few of them give the possibility to costumize data models and structures. Most of them are highly devoted to a specified field, thus resulting poor in flexibility. Actually, a number of these platforms have been designed to deal either with complex neuroimaging data schemes or with distributed resources on data and computational sides, but usually they do not take into account the possibility of considering extensible data models to add new types of data as well.

SenseLab [15] is a project of Yale University for flexibly storing data and metadata to be displayed within a Web-based user interface. The approach to address these issues is named EAV/CR (Entity-Attribute-Value with Classes and Relationships) and combines object-oriented concepts with the basic EAV model. Specifically, an object in the database must belong to a particular class, its attributes are constrained based on its class, and relationships are set between data objects. An EAV/CR database is based on a relational database using mostly an EAV-based storage mechanism. Flexibility is taken into account just as regards tables to be created and/or classes to be added to tables.

The GridPACS system, developed at Ohio State University similarly provides a distributed data archive and utilizes Grid technology for implementing processing pipelines [16]. Nevertheless, it doesn’t address issues about extensibility in data models and is strictly devoted to managing medical images.

Few other data management systems are mostly aimed at allowing XML-based descriptions of relational databases. Among them, we may cite Silkroute [17] and DataServer [18]. But none of them, actually, neither consider complex data schemes for neuroscience applicatons nor address the issue related to the use of distributed data and computational resources.

However, starting from these previous experiences, a reference platform for managing multimodality neuroscience data has emerged in the last years. Having extensibility as its reason for being, the eXtensible Neuroimaging Archive Toolkit (XNAT) [19] is a powerful software platform designed to facilitate the management and maintenance of neuroimaging data and related information developed by the Neuroinformatics Research Group in collaboration with the National Institute of Health (NIH). XNAT enables users to access the archive through a secure web application which provides a number of quality control and productivity features, supports a wide variety of methods to upload data, allows users to inspect, validate, and process the data and extend the default data schema. Moreover, backup processes can be scheduled and security protocols can be enforced, user interactions with the data are logged and modifications to data can be recorded and validated. Also, common processing routines can be automated and the resulting measures can be captured back into the archive. Moreover, the XNAT framework relies heavily on XML and XML Schema for its data representation, security system, and generation of user interface content. For all the features mentioned above and because of its use of XML for building extensible data models, XNAT has been considered as a reference platform for our work. However, after a comprehensive evaluation, some constraints in the overall approach of XNAT have been proven to be impractical in our application. The major obstacles were related to the manipulation and extension of the data model, the low flexibility in data management and the difficulty to access distributed computing resources and storage facilities. In particular, as regards extensibility, XNAT still needs computer science skills to manually write XML schemes to add a new data type as well as the rebuilding of the database and the re-deployment of the webapp. Moreover, a Grid based deployment of applications is not considered within such a platform. Further details on the comparison between our and XNAT’s approaches will be given in the methods section. As a further improvement, the Human Imaging Database (HID), developed by the Biomedical Informatics Research Network (BIRN), provides an extensible data management system for clinical neuroimaging, accessible through a web-based user interface [20], operating in a distributed environment. In respect to XNAT, HID allows the federation of different databases and relies on a schema, based on the XCEDE schema described above, with abstract data types, thus providing more efficient expansions without modifying the schema itself. HID can also mantain a virtual directory system of local and remote files managed through the Storage Resource Broker.

### The Grid approach

Actually, to address issues about distributed resources regarding both storage and computation, the Grid approach has been a successful way to manage some specific challenges about complex experiments in the biomedical field [21]. These challenges include the management of storage resources using different access protocols, the authentication and authorization across systems using different identity management systems, uniform management policies across institutions having differing access requirements and the need of a wide-area-network access.

iRODS, the Integrated Rule-Oriented Data System, is a data Grid middleware system developed by the Data Intensive Cyber Environments research group. The iRODS system is a generic middleware that can be configured to implement any desired data management application based on a set of major components including a data Grid Architecture based on a client/server model controlling interactions with distributed storage and computational resources, a Metadata Catalog maintained in a database system for managing the attributes of data and state information generated by remote operations and a Rule Engine enforcing and executing adaptive Rules. iRods is distributed with an open source BSD license and has been chosen as the reference Grid middleware for our software environment. More reasons about the choice of iRODS as Grid middleware are explained in the Methods section.

## Methods

Actually, XNAT and HID approaches provide a very sound base for working on these issues and it is far from our intention to compare our collaborative environment to such powerful and widely adopted platforms. Nevertheless there is still room for improvements, as regards extensibility and customization, and, for some specific scenarios, more customizable tools are needed to meet specific users requirements. Our work has been mostly aimed at addressing the needs of small laboratories without any or little technical expertise. To this goal, non technical users should be enabled to create, extend and modify the whole data scheme, if possible through the same user friendly web interface used to fill in data. Also, especially for multidisciplinary and multicentre experiments, particular security and privacy policies have to be addressed in regard to the access to proprietary data and sensible clinical data. Moreover, the management of genetic data often requires an integrated access to public databases such as NCBI and Molgen. Finally, especially in experiments including genetics screenings, tools must be provided for managing specimens and samples in local storage fridges. Our platform tries to answer to these needs in order to put non technical researchers from small laboratories in control of data and samples during collaborative experiments.

The need of extensibility has been considered from two different points of view. The first one is bound to the possibility to easily customize and extend the experimental procedures in order to log each step of acquisition or analysis. This is achieved through a process-event model, a multipurpose taxonomic schema composed by two generic main objects: events and processes. The second one is related to the improvement of data flexibility. This aspect has been taken into account through the development of a methodology for the dynamic creation and use of data types and related metadata, based on the definition of a “meta” data model. This issue is critical in order not to constraint the repository to a set of predefined data but to make it easily extensible and applicable to different contexts, also making data immediately usable and integrated.

Finally, data integration aspects have been addressed by efficiently storing distributed samples, data and metadata and providing the repository application with an efficient dynamic interface designed to enable the user to both easily query the data depending on defined data types and view all the data of every patient in an integrated and simple way.

### The process-event structure

**Figure 1:**
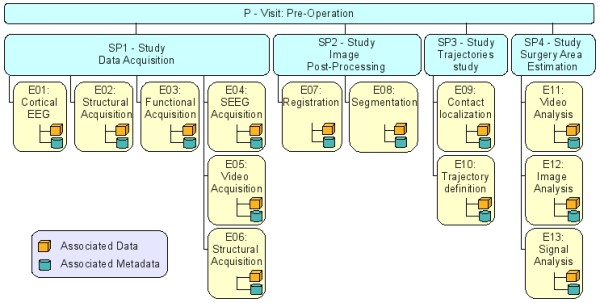
**Example of the XTENS process-events structure.** A complex process can include several studies, each composed of atomic events.

An event is defined as either any “atomic” operation that can be performed on patients, or any processing of data, or any other action related to the administration and management of the repository. If needed, it can contain correlations between data, metadata and, in addition, algorithms. Each event is associated with a process. The process is defined as a group of sequential events and/or sub-processes related to an activity, allowing the creation of a sort of hierarchical structure. Custom process-event types and their relationships can be defined, thus describing the taxonomy better fitting the needs of the application. An example of the process-events structure concerning a possible clinical scenario is shown in Figure 1. It is worth noting the existing relationships between processes (blue boxes) and events (yellow boxes) and the association of data and metadata to each specific event. A pre-surgical analysis sequence is considered. This is divided into different phases consisting, each, of different steps, ranging from the acquisition of data to their analysis. According to the described structure, a Pre-surgical Process (P) can be considered as a top level process composed by different sequential subprocesses: (SP1) Data Acquisition, (SP2) Image Post-Processing, (SP3) Trajectories study and (SP4) Surgery Area Estimation. Each of these sub-processes represents a specific part of the main process and is composed by a number of events, each of them connected to the related data and metadata. It is also worth noting that the whole process can be easily modified to fit either changes in the analysis sequence or different requirements for another case study, just by changing or adding new events, creating new processes composed of different events or combining existing processes and events. The defined process-event taxonomy can be used to store the information about each step in the process and the related data and metadata, thus allowing the definition of a detailed time line of performed operations. This can also improve the repeatability of experiments by providing both a detailed recording of the analysis process and a complete description of relationships between data and actions.

The XCEDE model has been tailored to fit this process/events model in a very simple and efficient way. As shown in Figure 1, the visit and study elements have been associated to process entities with a father-son relationship while episodes and acquisitions have been collapsed into events.

### The data model

The analysis of the state of the art pointed out that just a few data models/repositories have the ability to add new data types. However, in fact, in most cases, as their databases are based on the already defined data, they require the rebuilding of the database each time a new type of data is added.

For example, the procedure to add a new data type in XNAT requires the intervention of a specialist in order to: 

∙ Create an XML Schema

∙ Add the schema to the project

∙ Run the update script (bin/update.sh or bin/update.bat)

∙ Update the database

∙ Re-Deploy the webapp

∙ Setup XNAT security to allow access to the new data types

The proposed approach solves these issues providing a new methodology for the data and related metadata management to make the repository easier to use, more dynamic and therefore improving the overall extensibility and customization. This is based on the definition of a “meta” data model enabling the user to build his own data types independently from the application context. With this approach, in order to add a new data type, a user (even a clinical one) has just to: 

∙ Open a web page on the browser and build the data type structure using drop down lists and other simple controls

∙ Save the data type, and eventually assign the new data type only to a given set of users/groups

∙ Use the data type directly (without the need to restart the application or perform any other setup)

The presented approach, however, does not preclude the use of existing and standard data models. Indeed, through the development of suitable wrappers, it is possible to convert data into a standard compatible format as well as to create types of data from an existing data model. Furthermore, the presented model is general enough to be also extended in order to describe other entities like experiment descriptions.

A “data type” is identified as a minimum set of information describing a data instance (i.e., the set of records associated to a clinical study, the parameters for a particular biomedical image) that may or may not be associated to physical files. As an example, clinical data can be defined as datatype but are not associated to a file, unlike MRI data.

Each data type is described by an XML metadata schema associated to XSD and XSL files file to define, respectively, its structure and display. The XSD and the XSL adopted for transformations are the same for all XML files. From XML files, DHTML web forms are built, using XSL transformations. The XML can be stored in MySQL or in other SQL databases. In this case, an XML file URL can result not in a physical file but in a query to the DB (this is transparent to the component requiring the XML). This can be considered as a caching mechanism.

The XML representation of a data type metadata is divided into two main sections: a header, containing general information about the schema, and the metadata description, representing the detailed description of the information. The metadata description is composed by one or more groups of information each composed by attributes, loops and their combinations. An attribute defines a single metadata and is composed by different parameters and subelements. The formers describe the information related to the typology of the attribute (type, whether it is required, etc.). Subelements, instead, describe what the attribute represents: they include name, value or possible values and references to existing ontology definitions. Actually, through the interface used to build data types, it is possible to choose an ontology among those defined within the platform. Once an ontology has been chosen, it is temporarily loaded within the system and its terms are made available to support users in the attributes’ name definition. This is done thanks to a “suggest” mechanism. In fact, whenever a user digits the name field, an ontology based suggestion will appear according to the written text. This approach is very important in order to build data types using standard annotations and to make them being easily integrated with external data sources.

The loop is probably the most relevant improvement with respect to existing data models. Usually, when modelling clinical information, the information model (in terms of number of attributes) is clear but the amount of information (the number of occurences) that will characterize the specific data to be recorded is not. This is the case, for example, of reagents in microarray studies: ”reagent” is the information to be recorded, but for different microarray studies, different types of reagents can be used. Therefore, the information to be recorded under the “reagent” metadata is in fact characterized by description, concentration and amount for every used reagent. A similar issue in clinical studies arises describing risk factors: the presence of a risk factor is the information, and it can be modeled with details on every sub-factor presence and relevance (smoke, alchool, sedentary job).

### Overall system architecture

**Figure 2:**
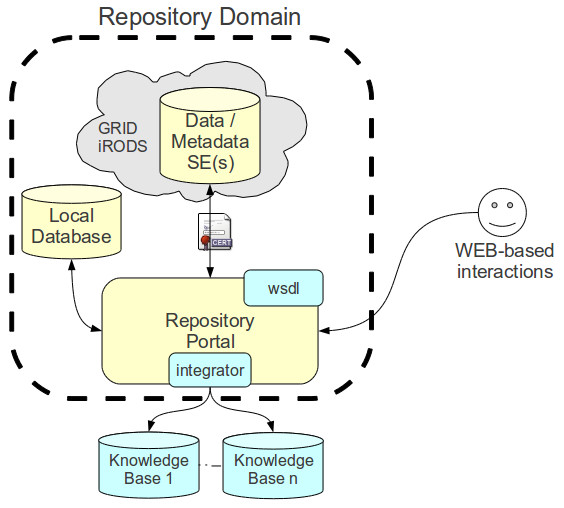
**XTENS overall architecture.** The repository is accessed through a web portal interacting both with a local database for patient metadata and with a Grid based Storage Element.

The main components of the Repository are (Figure 2): 

∙ the Repository portal: it provides a web interface and allows users to access and manage database requests. It is hosted by a Linux/Unix server environment;

∙ the Database: it hosts all the information about projects, subjects, metadata, etc.;

∙ the Grid Storage: it contains all data files;

Two important aspects are related to authentication and software as a service issues. As regards authentication, users and system administrators authenticate to the system using an existing LDAP or a database account available on the server infrastructure. The access is via web browser without any client installation and in a secure way through the HTTPS (secure HTTP) protocol. When a user needs to access the repository resources he has to authenticate himself through the web portal interface using his username and password. Each user is associated to Access Control Lists in order to guarantee security and auditing. System administrators are able to define different groups of users associated with different access permission to different pages and functions of the repository. This way, users can see only a subset of pages and perform a limited number of different actions (depending on their role in the project). The Web based SaaS (Software as a Service) approach has been preferred because the backend hardware can be scaled up enough to satisfy the user needs without requiring users to implement their own infrastructure. Moreover, this allows, at any moment, to migrate from a standard hardware infrastructure to a service based one, e.g. using the Grid or other kind of on demand IT services.

### The repository portal

The repository portal is designed to make the storage and the navigation of data and information easy, through a simple and transparent web interface. It is a Java 2 Enterprise Edition (J2EE) web application based on several existing open source tools for the development of web applications. The basis of the portal consists in a framework that relies on an Apache Tomcat web application container [22]. It incorporates a database interface layer built through iBATIS, a persistence framework which automates the mapping between SQL databases and objects in Java [23]. To provide users with highly interactive interfaces, some components are designed using the AJAX (Asynchronous Javascript and XML) programming technique. Messages are exchanged in XML or JSON (JavaScript Object Notation) [24] format wherever possible. Also, wherever possible, XSL transformations (to transform XML data into human readable HTML pages) are performed [25].

This component represents the main access point to all the functionalities available through the overall integration platform, and exposes both user and administrator interfaces. Administrators are able to control users’ access by creating groups and their association with pages and functions, define processes (visits and studies), events and all their relationships, define new data types and related metadata, associate them with the related events and manage available ontologies. Normal users, according to their assigned permissions, are able to insert new data, retrieve patients’ information and view all the related data, download stored data, explore visits, studies and their interconnection and all the related events, data and metadata to have a global picture.

As an additional feature, in order to make the insertion of metadata easier, an automated approach is available for some predefined datatypes like MRI, fMRI, PET and SPECT. Using libraries like Java dcm4che [26], the portal can automatically extract metadata contained within uploaded data files and incorporate them correctly in the database, creating associated events depending on image modality. Such as automatic procedure permits to avoid human errors and provides a further file type checking before the uploading. If needed, a visualization tool can be made available within the portal interface in order to allow users to interact with neuroimages through a remote visualization service. This is made possible by using a client-side application that uses the VNC protocol to connect to a sharable work session that is running server-side, with a significant speed up of diagnostic processes.

### The database

The Repository is based on a MySQL database. The database design has been a crucial part of the repository development. In fact this component is fundamental in order to make the repository highly flexible and easily extensible. The core of the database is formed by the two previously described entities: processes and events and their relationship to data and metadata. The information inside the data table represents the data inserted in the repository. These data can be associated with one o more files, thus keeping the association with one or more file entities accordingly to their datatype. The File table contains the URIs of all the stored files. The repository can be configured to store the metadata totally or partially within the database. In this latter case, the metadata are stored as XML descriptions inside the data table, to display the data in a rapid and dynamic way using XSLT Transformations and as records of specific metadata tables, to perform complex queries in an easier way.

### The Grid middleware

A crucial aspect of the the repository design is related to the choice of the Grid middleware used to build the underlying infrastructure. The storage subsystem has been built around the iRods tool, the successor of SRB (Storage Resource Broker, by the San Diego Supercomputing Center) [27]. iRods has been chosen, among others (e.g. gLlite Storage Element subcomponent) because it allows to build a federated and distributed data storage system without the need of central components. In fact, the gLite middleware provides a storage component with many features (command line and Java APIs, integration with x.509 certificates, integration with key-stores for crypted data storage) and, in a separate component, a metadata catalog (AMGA). However, it requires almost dedicated servers, with a specific Linux flavour on top and a bit of infrastructure (resource brokers) to deal with, thus requiring (almost partially) specific technical staff. Therefore, as our platform is mostly aimed at small laboratories mostly focused on biomedical skills and with a low level hardware infrastructure, the choice of iRODS as Grid storage middleware has been preferred despite the advanced features of gLite. Moreover, in the planned experiment, the requested amount of storage was really basic and the storage infrastructure had not to be shared on a public Grid virtual organization, thus suggesting a simpler solution. Finally, we also decided to use iRODS as the basis of the storage architecture of the repository because it permits the use of heterogeneous storage resources and allows the creation of microservices and rules to easily perform operations on the stored data and metadata. Anyway, it is worth mentioning that iRODS can be interoperable with the gLite Storage Resource Manager (SRM) [28] interface thanks to the work carried out at the Academia Sinica Grid Computing group, Taiwan [29].

### The samples manager

The system described in this paper has been realized with the main goal of providing a tool for everyday activity of data collection and search, with a really pragmatic and practice oriented perspective. One of the peculiarity of our multi-centre study is that blood samples taken in Genoa, Italy have to be sent to the Health Science Center, Lubbock, Texas, for the genetic analysis to be carried out. Samples have to be stored in freezers, as different analyses are carried out over time. To streamline the process of storing and retrieving samples, an ad-hoc Web application has been developed, fully integrated in the platform. The application allows biologists to register the actual location of the sample in the freezer benches (-80C and -45C), also allowing technicians to configure graphically the available racks and slots in their freezers. When they put a blood sample (or a processed DNA/RNA sample) in the freezer, they can input the coordinates of the sample, from the freezer identification number down to the x-y coordinate in the sample box, simply by clicking on the application interface. They can also save the courier mailing number and a reference to the patient. This way, a sample can be retrieved at any time and is unequivocally associated with other patient data.

## Results

### The scenario

Our platform is being tested within a research project currently carried out through an international collaborative effort between the Laboratory of Molecular Neurogenetics at the Texas Tech University Health Sciences Center (TTUHSC) and two Departments at the University of Genoa, Italy: the Department of Neuroscience, Ophthalmology and Genetics (DINOG) - Section of Clinical Neurophysiology and the Department of Computer Science, Bioengineering, Robotics, and Systems Engineering (DIBRIS).

The aim of the study is to develop criteria for the early diagnosis of Alzheimer’s disease (AD) and algorithms to predict the progression of the disease, by combining neuropsychological tests, imaging methods, genetic tests and biomarker screening. The results of the implementation of the described platform within such a scenario are presented below just as an assessment of the feasibility of our approach in case of small multi-modality and multi-level experiments. Even though the architecture has been explicitely thought for facing extensibility issues, not only in terms of data model but also in terms of larger datasets and more distributed hardware infrastructure, the scalability of our approach has not been tested yet, due to the limited dimension of the experiment. However, a first set of 20 patients with their clinical data and brain scans, as well as results from the related genetic screening, have been inserted, so far, through our platform. We plan to insert about 50 patients a year due to the strict policies adopted for patient eligibility.

The steps identified within the collaboration in order to achieve the project goals are as follows: 

∙ Identify and enroll subjects: DINOG recruits patients with the diagnosis of single- or multi-domain aMCI (amnestic Mild Cognitive Impairment), Alzheimer’s disease and normal controls of a defined geographical area (Genoa, northern Italy). The clinical evaluation of patients is based on an initial interview, a complete general medical examination and a battery of neuropsychological tests. Further, MRI and fluorine-18 (F-18) fluorodeoxyglucose-PET scans are performed.

∙ Perform genetic and biomarkers screening: TTUHSC investigates the genetic makeup and the genetic markers of the study subjects to identify the intrinsic factors involved in the pathogenesis of dementia.

∙ Collect data: DIBRIS and TTUHSC collect and store, over time, the results of clinical investigations and brain scans, together with the genetic and biomarkers screening in the multimodal and multiscale repository.

**Figure 3:**
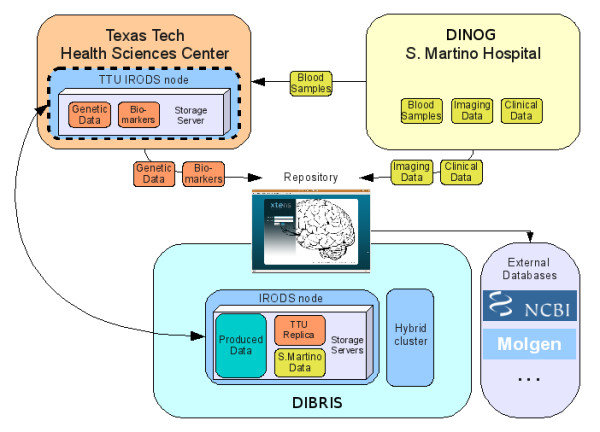
**Partner roles and contribution summary.** DINOG manages neurophysiological and neuroimaging data, TTHSC manages genetic data, DIBRIS manage the software and hardware infrastructure.

∙ Analyze multimodal data: specific algorithms will be developed, in collaboration with physicians and geneticists, to analyze and combine the acquired information. The generated algorithms will be valuable tools for the early detection of disease and the prediction of disease progression. Figure 3 summarize the overall collaboration with roles and contributions of each partner.

After neurophysiologists identified subjects eligible for the study, they have inserted the related personal data into the system together with clinical data, neuropsychological tests and neuro imaging data. Neuroimaging data are associated to files, while clinical data and neuropsychological tests are simple data without any file association. Data have been fully anonymized (and also defaced in case of morphological images) and cannot be linked in any way to patient’s names. The link between data in the repository and patients identities is done by using unique and anonymous identifiers managed only by clinicians. This solution was one of the project requirements and satisfies the guidelines authors have been asked to follow about data privacy.

Then, neurologists have provided the blood samples drawn from the subjects. In order to make samples management easy, a specific interface has been created to add samples, associate them with the corresponding patients and store the related information. Finally, these samples have been collected, frozen and sent, using couriers, to the Texas Tech group to generate genetic data.

On their turn, at first, geneticists have configured the genetic screening setup by inserting information about the genes to be screened, their exons and their relevant known variants. This configuration has been performed through the integration with online resources such as NCBI (National Center for Biotechnology Information) [30] and Molgen (Alzheimer Disease and Frontotemporal Dementia Mutation Database) [31] in order to retrieve and/or compare information. Users can directly view/access both the NCBI and the Molgen sites, the first pointing to the gene page and the second pointing to the gene variants section, in order to retrieve and store the correct and complete information within the repository. This is a very powerful tool to integrate the information coming from public databases into the repository.

A second step has been related to the insertion of new data obtained by genetic screening on the patients samples. This has been carried out through a devoted section in the user interface.

Finally, geneticists have completed the samples management process by inserting information about the delivery and storage of samples. In regard to this last aspect, the platform includes an application described in methods section, able to describe the real configuration of the storage fridges in each laboratory, thus allowing users to put and move samples in such structures and to track them over time. By means of such an integration, authorized users are able to manage samples and their storage information through a unique application.

In general, the access to patient information can be restricted because of privacy policies and only authorized users can insert, view or modify data. Users can view more or less information according to their permissions: for example, geneticists users are not allowed to see patients personal data but only their clinical data. This is another feature able to match important requirements for these kinds of experiments usally based on strict privacy policies.

As regards data mining, patient-related variables like diagnosis, sex and birth date can be combined with data information in order to compose specific queries. In fact, it is possible to add many different conditions in a dynamic way. As such, by choosing any of the defined data types, the corresponding “field” parameter is automatically filled with its specific defined attributes. Once a field has been selected, it is sufficient to insert the desired “value” parameter to complete the condition. This method makes it possible to query patients having data with one or more specific metadata attributes for any arbitrary combination of their values. It is therefore easy to imagine how to build complex queries choosing and combining patient related values (diagnosis and sex) with complex conditions on specific data and metadata. For example a query might be built to retrieve all male Amnestic MCI patients that are smokers, having variants for MAPT gene and one or more MRI data. After the query has been performed and patients have been retrieved, users can have a quick overview of all their data using an interface divided into three main sections. The first one is the same used to build the query as described above and can be used to refine the query. The second section contains the list of retrieved patients. For each selected patient in the list a third section shows a synthetic view of the related data. Each field can be further examined in details.

From the architectural point of view, a Data Grid paradigm allows keeping a unified control on the access and storage of data in resources geografically distributed among different centers. This can be done through secure protocols, also hiding the real infrastructure from users. In details, a main iRODS server has been installed within the DIBRIS infrastructure and further iRODS storage resources are located at Texas Tech University (Figure 3).

### Testing the platform

**Figure 4:**
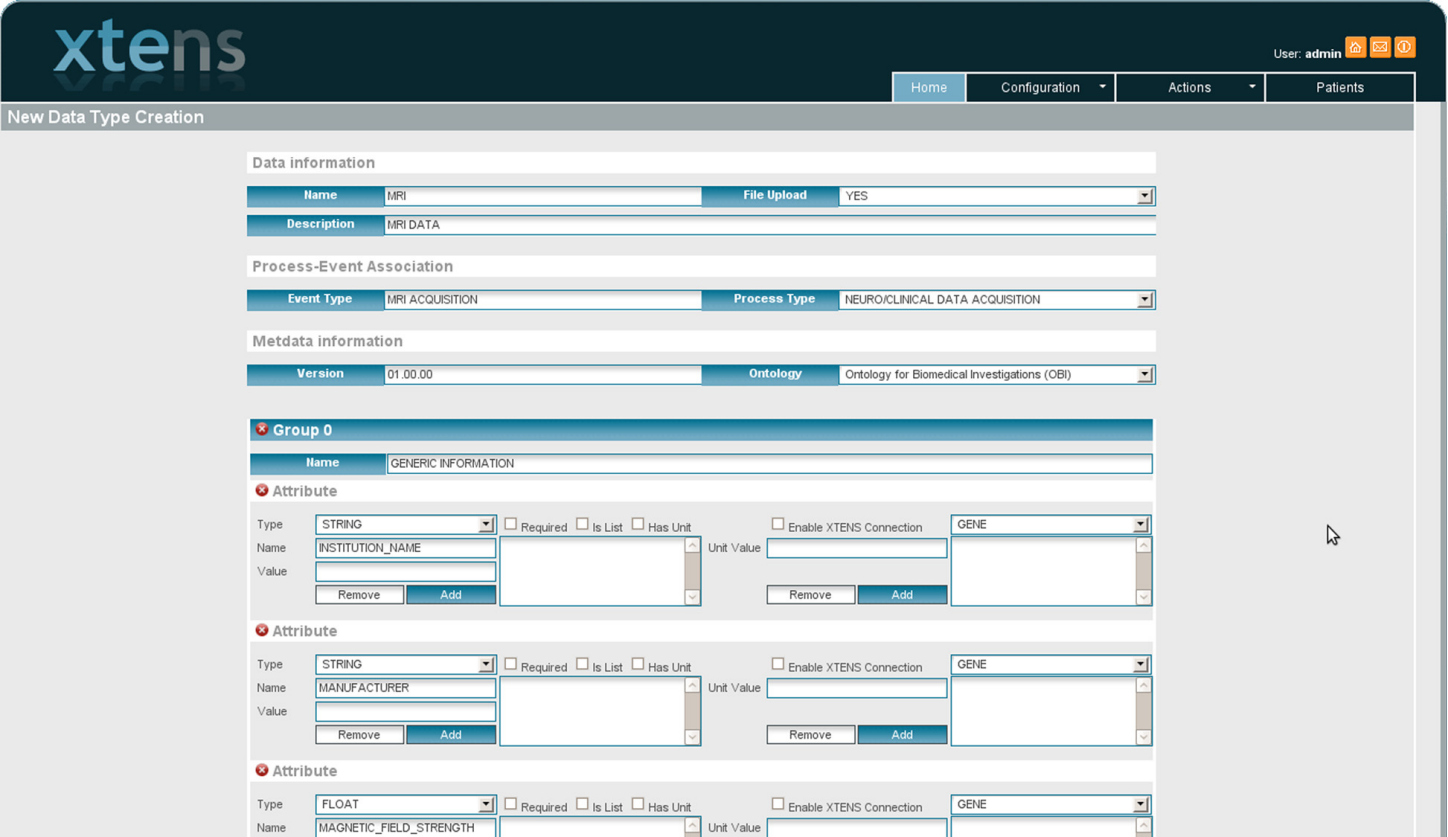
**Interface to define new data types.** The new data type is highly configurable thanks to both pre-defined and custom attributes.

Both clinicians and genetists were then asked to test the functionality of our platform in inserting and retrieving data and metadata. Four different scenarios were defined for testing the usability and the effectiveness of the Xtens environment. The first scenario involved the insertion by clinicians of the clinical history, results of general medical and neurological examinations, neuropsychological test scores and neuroimaging data including structural MRI and FDG-PET for a 74-year old woman with just mild impairment in memory test, but not demented. In the second scenario, the group of geneticists had to access, through an action toolbar, a page within the repository that is specifically set to support and facilitate the record of genetic data. Candidate genes can be selected from a drop-down menu and known single nucleotide polymorphisms can be entered manually or downloaded from the most relevant field specific websites (NCBI and Molgen) for each selected gene. Consequently, the geneticists are enabled to match their original findings with the ones from Ncbi and Molgen and to catalogue them as known or novel variants or mutations. As for the third scenario, the system was asked to list all subjects scoring less than 6 on the delayed recall measure of the Rey Auditory Learning Verbal Test, the standard test in use to detect short-term memory impairment. These subjects are more likely to be affected by a neurodegenerative disease than subjects with deficit in other cognitive domains. The results were displayed in a system window listing the identity number of those subjects whose information could be easily accessed by simply clicking on the identity number itself. The latter scenario was about the definition of a new data type concerning a generic serum biomarker in order to test the user friendliness of extending the data model. Users can either modify already defined processes, by selecting them from a list, or create new ones in an easy way, by means of the interface shown in Figure 4. Once a data type was built, related XML file and database structures are automatically created and stored as described before, thus completely hiding the underlying structures and layers from users. After the data scheme was defined, users started filling in their data into the repository. However, the schema can still be modified and extended after the insertion of data without any need of rebuilding the database.

From such usability validation some qualitative results may be evidenced. 

∙ Each individual with access rights (clinicians, geneticists or other potential users) was able log onto the repository and easily enter the data they have collected or generated.

∙ Each user had a correct full or partial access permission depending on their local ethical approval for access to patients’ confidential data.

∙ No critical issues emerged about the data workflow and the sequence of data input. Data input can be performed incrementally by resuming already active sessions when new data are available. All users found the user interface very effective.

∙ When genetic data were involved, the repository provided tools for managing specimens and samples in local storage fridges.

∙ The repository allowed to retrieve specific or targeted data or multiple data in association with other parts of the repository.

∙ Specifically, investigators were able perform searches within the repository in order to retrieve all clinical and genetic data of a part of the study population (i.e. based on diagnosis, or age, or sex or all together or based on the genetic findings in one specific candidate gene). Not only entering genetic data was easy and fast, but there was the opportunity to correlate different types of data at once.

∙ Geneticists and clinicians can use this repository to find a correlation between the genetic findings in patients who manifest certain clinical features. This will ultimately help to classify disease entities more precisely based on the genetic variations.

**Figure 5:**
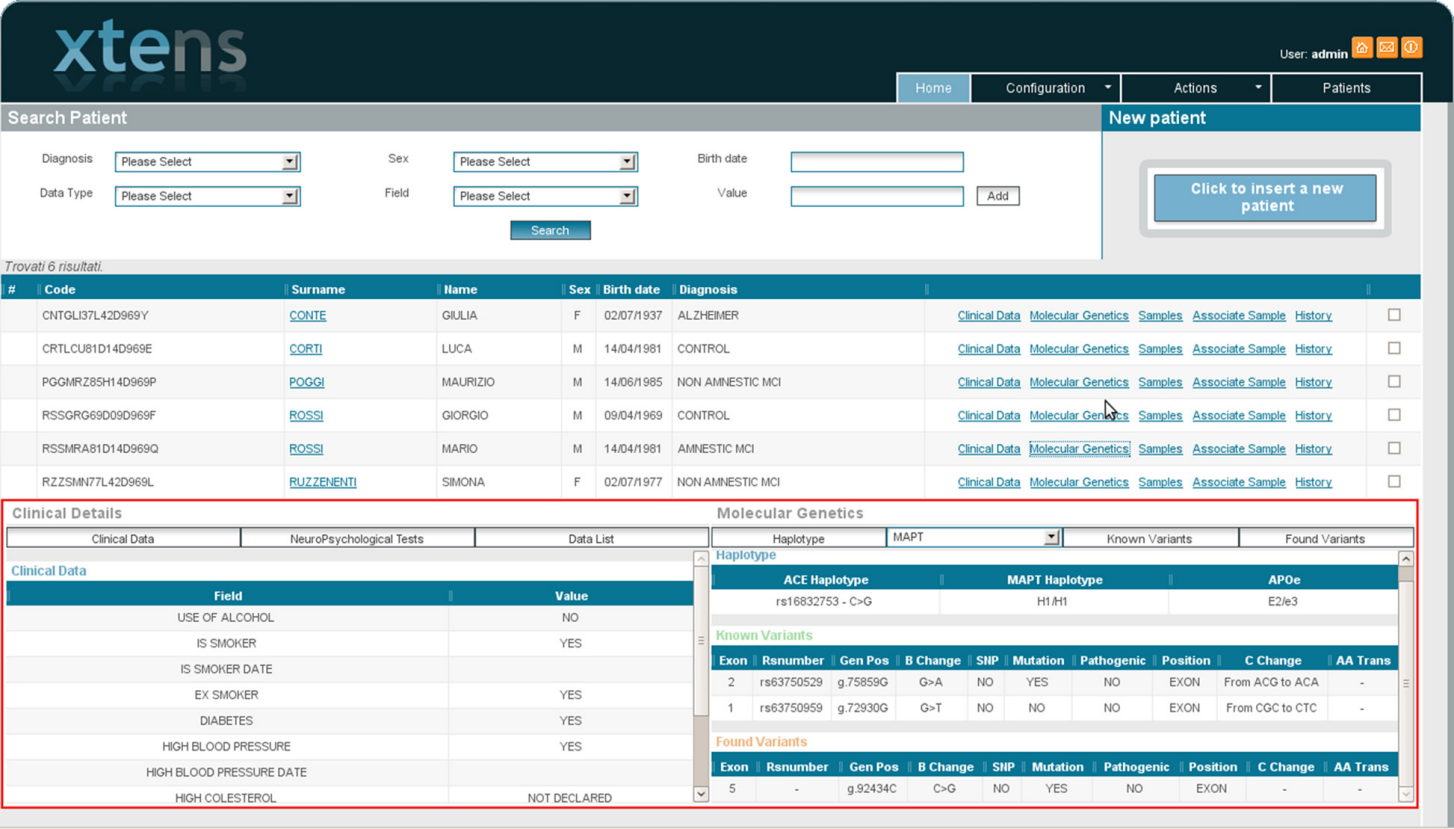
**Interface to view and browse data of the patients.** The red box highlights the visual integration between genetic and clinical data allowing an immediate overview.

∙ A vast variety of data are visualized all together on the same screen (Figure 5) helping in having an overview on whole data set, in interpreting data and in identifying novel opportunities and needs for one’s specific study. However, some users suggested to give the possibility of splitting different kinds of data in different windows.

∙ The extension of the data model through the definition of custom data types can be easily performed by users. The new data type was highly configurable thanks to a user friendly interface. No other operations were requested to users and the complexity of changes in the data structure was completely hidden from them.

A more quantitative evaluation, together with a scalability report, is planned to be presented in a further paper when the deployment of the repository will be completed.

## Discussion

A web repository based on a dinamically extensible data model has been developed to collect multimodal and multiscale data and to access patient studies.

Through the definition of a “meta” data model, aspects related to data and metadata management have been handled. Particular attention was, in fact, given to issues related to the definition and development of a methodology to create and use dynamic data types and their metadata through appropriate XML schemas. This way, the repository has not been limited to a set of predefined data but has been made easily extensible and applicable to different contexts, being thus data readily usable and integrated. The dynamical extension and modification of data types have been made possible also for non technical users through a user friendly web interface fully integrated in the platform and very similar to the one used to fill in data. This approach is able to hide the complexity of XML structures and functions from users with biomedical skills and is a very important feature for small laboratories focused on medical and genetics tasks. The adopted methodology is also able to automatically generate interfaces to enter data and metadata by using XSL transformations, and to perform complex queries composed on the basis of stored data and metadata. Extensibility issues have been also managed through the definition and implementation of a process-event model, a multipurpose taxonomic schema designed to easily customize and extend the experimental procedures in order to log each step of acquisitions or analyses. Such a model fits into the neuroscience context due to the adoption of concepts defined within the XCEDE model (project, visits, studies, episodes, acquisition).

Also, we have addressed possible particular security and privacy policies regarding the access to proprietary data and sensible clinical data. This has been achieved by a fully customization of user permissions, thus producing a clear association of users to data and services they are allowed to access.

Moreover, an integrated access to public databases such as NCBI and Molgen has been provided within our platform, as an aid to geneticists during the configuration of the genetic screening setup for inserting information about the genes to be screened, their exons and their relevant known variants.

Finally, due to requests arising especially in experiments including genetics screenings, an ad-hoc Web application has been developed, fully integrated in the platform, allowing biologists to track the actual location of samples in the freezer benches and to associate a sample to the related patient. It also allows technicians to reproduce graphically the real structure of racks and slots in their freezers and put samples in the right places.

Moreover, a Grid approach has been considered and implemented in order to manage distributed, heterogeneous data and information, improve security policies and facilitate collaborative work. Furthermore, the Grid paradigm enables a safe access to data stored on distributed resources by using an X.509 based secure access. Finally, the whole developed architecture and platform have been deployed and customized in order to fit the identified use case. As regards the presented scenario, data and results coming from clinical investigations and brain scans, together with data from genetic screening are currently being collected and stored over time within the repository.

Even though an accurate technical test has been already performed on our platform in order to check all the functionalities provided to users, a more rigorous and systematic evaluation of the ability of the platform to address robustness and scalability issues is still in progress. A usability validation was performed to check whether user requests have been fulfilled by our platform and its results show a good satisfaction. In particular, some specific services provided through the user interface were largely appreciated. Findings concerning more specifically the clinical issues faced in the experiment will be presented in a next paper.

As a final consideration, we would like to point out that our platform is not intended for large, more general neuroscience experiments but, rather, for relatively small projects with specific needs about extensibility and customization. This is to say that some general issues faced by widely adopted platforms, like XNAT and HID, have not been considered in our work. Therefore, some limitations are present in our approach as regards, for instance, either automatic procedures for data encription and validation or database federation. Nevertheless, on one hand, data have been fully anonymized and their validation has been achieved through strict protocols concerning data acquisition and, on the other hand, federation of databases is not a requested option in the type of projects which we are dealing with.

## Conclusions

As future developments of the presented platform, next steps will be about: (i) the identification of algorithms for data analysis and visualization tools; (ii) their implementation/integration within the provided infrastructure by creating services able to analyse and view data through remote computational and visualization resources.

Afterwards, a possible future development is related to the improvement of the process-event model. In fact, such methodology is highly flexible and its conception can be developed beyond a taxonomic schema to describe procedures. The related processes and events could be defined depending on the analyses to be performed and the new results data to be stored.

The described structured information might also be used as a reference to compose workflows of analysis. The planned improvement is related to the possibility of associating services to events. This would enable users to create simple or complex pipelines involving stored data and defined processes and events, convert the workflow in a standard format and execute it using an integrated workflow manager.

Finally, specific algorithms to analyze and combine the acquired information might be developed and exposed as services.

## Competing interests

Authors have no competing interests as regards issues considered in the paper.

## Authors’ contributions

LC participated in the design of the system, carried out the development of the code and helped to draft the manuscript, IP participated in the design of the system, carried out the development of the code and helped to draft the manuscript, AS participated in the design of the system and helped to draft the manuscript, PM participated in the definition of genetics data types and structures and helped to draft the manuscript, RF participated in the definition of genetics data types and structures and helped to draft the manuscript, FN participated in the definition of neurophysiological data types and structures and helped to draft the manuscript, MFerrara participated in the definition of neurophysiological data types and structures and helped to draft the manuscript, GA helped to develop the code and helped to draft the manuscript, MFato participated in the design of the system and helped to draft the manuscript. All authors read and approved the final manuscript.

## Pre-publication history

The pre-publication history for this paper can be accessed here:

http://www.biomedcentral.com/1472-6947/12/115/prepub

## References

[B1] International Neuroinformatics Coordination Facility [http://www.incf.org/]

[B2] Amari S Beltrame F Bjaalie J Dalkara T Schutter ED Egan G Goddard N Gonzalez C Grillner S Herz A Hoffmann K Jaaskelainen I Koslow S Lee S Matthiessen L Miller P Silva FD Novak M Ravindranath V Ritz R Ruotsalainen U Sebestra V Subramaniam S Tang Y Toga A Usui S Pelt JV Verschure P Willshaw D Wrobel A Neuroinformatics: the integration of shared databases and tools towards integrative neuroscience J Integr Neurosci 2002 1 2 117 128 10.1142/S0219635202000128 15011281

[B3] Phan J Quo C Wang M Functional genomics and proteomics in the clinical neurosciences: data mining and bioinformatics Progress in brain research 2006 158 83 108 17027692 10.1016/S0079-6123(06)58004-5

[B4] XML-Based Clinical Experiment Data Exchange Schema (XCEDE) [ http://www.xcede.org]

[B5] Biomedical Informatics Research Network (BIRN) [ http://www.birncommunity.org/]

[B6] Keator DB Grethe JS Marcus D Ozyurt B Gadde S Murphy S Pieper S Greve D Notestine R Bockholt HJ Papadopoulos PM A National Human Neuroimaging Collaboratory Enabled by the Biomedical Informatics Research Network (BIRN) IEEE Transactions on Information Technology in Biomedicine 2008 12 2 162 172 [ http://dblp.uni-trier.de/db/journals/titb/titb12.html#KeatorGMOGMPGNBP08] 18348946 10.1109/TITB.2008.917893 PMC2763494

[B7] Jones A Miller M Aebersold R Apweiler R Ball C Brazma A DeGreef J Hardy N Hermjakob H Hubbard S Hussey P Igra M Jenkins H Julian R Laursen K Oliver S Paton N Sansone S Sarkans U Stoeckert C Taylor C Whetzel P White J Spellman P Pizarro A The Functional Genomics Experiment model (FuGE): an extensible framework for standards in functional genomics Nat Biotech 2007 25 10 1127 1133 [ http://dx.doi.org/10.1038/nbt1347] 10.1038/nbt1347 17921998

[B8] Perez-Rey D Maojo V Garcia-Remesal M Alonso-Calvo R Biomedical ontologies in post-genomic information systems Fourth IEEE Symposium on Bioinformatics and Bioengineering BIBE 2004. Proceedings 2004 207 214

[B9] The Open Biomedical Ontologies [ http://obo.sourceforge.net]

[B10] Smith B Shburner M Rosse C Bard J Bug W Ceusters W Goldberg L Eilbeck K Ireland A Mungall C Leontis N Rocca-Serra P Ruttenberg A Sansone S Scheuermann R Shah N Whetzel P Lewis S The OBO Foundry: coordinated evolution of ontologies to support biomedical data integration Nat Biotech 2007 25 11 1251 1255 [ http://dx.doi.org/10.1038/nbt1346] 10.1038/nbt1346 PMC2814061 17989687

[B11] The Ontology for Biomedical Investigation [ http://obi.sourceforge.net]

[B12] Whetzel PL Brinkman RR Causton HC Fan L Field D Fostel J Fragoso G Gray T Heiskanen M Hernandez-Boussard T Morrison N Parkinson H Rocca-Serra P Sansone SA Schober D Smith B Stevens R Stoeckert CJ Jr CT White J Wood A Group FW Development of FuGO: An Ontology for Functional Genomics Investigations OMICS 2006 10 2 199 204 10.1089/omi.2006.10.199 16901226 PMC2783628

[B13] The Sequence Ontology [ http://www.sequenceontology.org]

[B14] Eilbeck K Lewis SE Sequence ontology annotation guide Comp Funct Genomics 2004 5 8 642 7 10.1002/cfg.446 18629179 PMC2447471

[B15] Marenco L Tosches T Crasto C Shepherd G Miller P Nadkarni P Achieving evolvable web-database bioscience applications using the EAV/CR framework: recent advances J Am Med Inform Assoc 2003 10 444 453 10.1197/jamia.M1303 12807806 PMC212781

[B16] Hastings S Oster S Langella S Kurc T Pan T Catalyurek U Saltz J A Grid-Based Image Archival and Analysis System J Am Med Inform Assoc 2005 12 3 286 295 10.1197/jamia.M1698 15684129 PMC1090459

[B17] Fernández M Kadiyska Y Suciu D Morishima A Tan W SilkRoute: A framework for publishing relational data in XML ACM Transactions on Database Systems 2002 27 4 438 493 10.1145/582410.582413

[B18] Bui A Weinger G Barretta S Dionisio J Kangarloo H An XML Gateway to Patient Data for Medical Research Applications Annals New York Academy Sciences 2002 980 236 246 10.1111/j.1749-6632.2002.tb04900.x 12594093

[B19] Marcus D Olsen T Ramaratnam M Buckner R The Extensible Neuroimaging Archive Toolkit: an informatics platform for managing, exploring, and sharing neuroimaging data Neuroinformatics 2007 5 11 34 17426351 10.1385/ni:5:1:11

[B20] Ozyurt WDKDBPSGBGG BI Grethe JS Federated Web-accessible clinical data management within an extensible neuroimaging database Neuroinformatics 2010 8 4 231 249 10.1007/s12021-010-9078-6 20567938 PMC2974931

[B21] Maojo V Tsiknakis M Biomedical informatics and healthGRIDs: a European perspective IEEE Eng Med Biol Mag 2007 26 3 34 41 17549918 10.1109/memb.2007.364927

[B22] Apache Tomcat web application container [ http://tomcat.apache.org/]

[B23] iBATIS [ http://ibatis.apache.org/]

[B24] JSON (JavaScript Object Notation) [ http://www.json.org/]

[B25] XSL Transformation [ http://www.w3.org/TR/xslt]

[B26] Warnock M Toland C Evans D BWallace Nagy P Benefits of using the DCM4CHE DICOM archive J Digit Imaging 2007 20 suppl1 125 129 17917780 10.1007/s10278-007-9064-1 PMC2039778

[B27] iRODS White Paper [ https://www.irods.org/pubs/DICE_iRODS_White_Paper-08.pdf]

[B28] Storage Resource Manager Working Group [ http://sdm.lbl.gov/srm-wg/]

[B29] Ueng W Chen H Grid Interoperation: SRM-iRODS interface Development Proceedings International Symposium on Grids and Clouds (ISGC 2011), 19-25 March 2011 Taipei, Taiwan

[B30] National Center for Biotechnology Information (NCBI) [ http://www.ncbi.nlm.nih.gov/]

[B31] Molgen (Alzheimer Disease and Frontotemporal Dementia Mutation Database) [ http://www.molgen.ua.ac.be/Admutations]

